# Gene Expression Profiles of Main Olfactory Epithelium in Adenylyl Cyclase 3 Knockout Mice

**DOI:** 10.3390/ijms161226107

**Published:** 2015-11-30

**Authors:** Zhenshan Wang, Yanfen Zhou, Yingtao Luo, Jing Zhang, Yunpeng Zhai, Dong Yang, Zhe Zhang, Yongchao Li, Daniel R. Storm, Runlin Z. Ma

**Affiliations:** 1 College of Life Science, Hebei University, Baoding 071002, China; zhou66913@163.com (Y.Z.); luoyingtao1987@sina.com (Y.L.); zhangjing_0521@sohu.com (J.Z.); zypkid1412@sina.com (Y.Z.); yangdong87@yahoo.com (D.Y.); zhangzhe_2005@126.com (Z.Z.); 2 Institute of Genetics and Developmental Biology, Chinese Academy of Sciences, Beijing 100101, China; liyongchao@genetics.ac.cn (Y.L.); rlma@genetics.ac.cn (R.Z.M.); 3 Department of Pharmacology, University of Washington, Seattle, WA 98195, USA; dstorm@u.washington.edu

**Keywords:** microarray analysis, differentially expressed genes, adenylyl cyclase 3, main olfactory epithelium

## Abstract

Adenylyl Cyclase 3 (*AC3*) plays an important role in the olfactory sensation-signaling pathway in mice. *AC3* deficiency leads to defects in olfaction. However, it is still unknown whether *AC3* deficiency affects gene expression or olfactory signal transduction pathways within the main olfactory epithelium (MOE). In this study, gene microarrays were used to screen differentially expressed genes in MOE from *AC3* knockout (*AC3*^−/−^) and wild-type (*AC3*^+/+^) mice. The differentially expressed genes identified were subjected to bioinformatic analysis and verified by qRT-PCR. Gene expression in the MOE from *AC3*^−/−^ mice was significantly altered, compared to *AC3*^+/+^ mice. Of the 41266 gene probes, 3379 had greater than 2-fold fold change in expression levels between *AC3*^−/−^ and *AC3*^+/+^ mice, accounting for 8% of the total gene probes. Of these genes, 1391 were up regulated, and 1988 were down regulated, including 425 olfactory receptor genes, 99 genes that are specifically expressed in the immature olfactory neurons, 305 genes that are specifically expressed in the mature olfactory neurons, and 155 genes that are involved in epigenetic regulation. Quantitative RT-PCR verification of the differentially expressed epigenetic regulation related genes, olfactory receptors, ion transporter related genes, neuron development and differentiation related genes, lipid metabolism and membrane protein transport *etc.* related genes showed that *P75NTR*, *Hinfp*, *Gadd45b*, and *Tet3* were significantly up-regulated, while *Olfr370*, *Olfr1414*, *Olfr1208*, *Golf*, *Faim2*, *Tsg101*, *Mapk10*, *Actl6b*, *H2BE*, *ATF5*, *Kirrrel2*, *OMP*, *Drd2* *etc.* were significantly down-regulated. In summary, *AC3* may play a role in proximal olfactory signaling and play a role in the regulation of differentially expressed genes in mouse MOE.

## 1. Introduction

Olfaction is important in the lives of both animals and humans. The main olfactory epithelium (MOE) is the primary olfactory organ in mammals for the perception of odor molecules [1]. Within the MOE, are layers of cells with the mature olfactory neurons on the top and the immature olfactory neurons on the bottom. The mature olfactory neurons have sensory cilia which express the primary signaling components for olfaction including odorant receptors [2], Golf [3], *AC3* [4] and the cyclic nucleotide gated ion channel (CNG channel) [5]. The main role of *AC3* is to catalyze the conversion of adenosine triphosphate (ATP) to cyclic adenosine monophosphate (cAMP) which then activates the CNG for exchange of calcium ions and chloride ion efflux, transmembrane protein (TMEM) channels, to cause membrane depolarization [6,7,8,9]. *AC3* deficient mice exhibit olfactory dysfunction, adult obesity and malfunction of smell related behaviors [4,10,11,12,13].

Previously, we have used Suppression Subtractive Hybridization (SSH) to analyze the molecular mechanism of *AC3* related olfaction disorder [14]. Wang *et al.*, reported that the activated transcription factor 5 (*ATF5*) interacts with the expression of *AC3* [15]. After knocking out *ATF5*, *AC3* expression decreased significantly and the differentiation of immature olfactory neurons into mature olfactory neurons was severely impaired, with the number of mature olfactory neurons also significantly decreased [15]. In addition, the expressions of genes associated with epigenetic regulation, including *LSD1* and *H2BE*, were also regulated by *AC3* [16,17,18]. These studies provided insight concerning the transcriptional role of *AC3* in olfaction. However, the expression of individual genes in an organism is the result of a network of regulatory genes. An accurate and comprehensive study on whether *AC3* inactivation might lead to changes in the expression of related gene groups in MOE has not been reported.

Microarrays have been widely used due to their broad detection range and accuracy. Mand *et al.*, have found, using microarrays, that the expression of the olfactory receptor genes, such as *Olfr156*, *Olfr382*, *Olfr1163*, *Olfr1219* and *Olfr1234* in the MOE of neonatal mice was significantly higher than those in the adult mice [19]; Sammeta *et al.* [20] have also used microarray chips to identify over 10000 genes that were expressed in mature olfactory neurons; hundreds of differentially expressed genes between mature and immature olfactory neurons have also been discovered using microarray chips [21]. The expression profile of the globose basal cells in MOE was analyzed using microarrays [22]. In addition, microarrays have also been applied to identify large numbers of genes expressed in the mouse MOE that are heavily involved in apoptosis, immune responses, cell cycle, transcription factors and genes that regulate cell proliferation, development and differentiation [23,24,25,26]. Using microarray chips to conduct comparative analyses of gene expression in the MOE from wild-type and cyclic nucleotide gated channel alpha 2 (*CNGA2*) knockout mice, Bennett *et al.* [27] found *CNGA2* regulated the expression of olfactory related genes. Kajimura *et al.* [28] has also conducted microarray hybridization analysis between the MOE in wild-type and *Klf* knockout mice. Their results showed that the expression of genes associated with cell adhesion, neurite growth, cytoskeleton dynamics, synaptic vesicles, transmission *etc.* in the MOE were all regulated by *Klf* [28]. These studies demonstrated that the combinational usage of the microarray and knockout mice is an effective strategy to study gene groups regulated by a target gene and to identify the mechanism of the regulation.

In order to identify whether gene expression associated with the network in MOE would be affected if *AC3* is deleted, in this study, we used MOE tissues from *AC3* knockout (*AC3*^−/−^) and wild-type mice (*AC3*^+/+^), screened for differentially expressed genes using microarrays, and further verified these results by bioinformatic analysis and qRT-PCR.

## 2. Results

### 2.1. Effect of AC3 Knockout on Gene Expression in Mouse MOE

To define the effects of *AC3* gene knockout on gene expression in the mouse MOE, the Agilent Mouse Genome 4 X 44K chip was used for hybridization. An Agilent Microarray Scanner platform was used to scan the hybridized chip. After comparison of all genes expressed in the MOE of *AC3*^−/−^ and *AC3*^+/+^ mice, we found that among the 41266 gene probes, 3379 had greater than a 2.0-fold change in expression levels between *AC3*^−/−^ and *AC3*^+/+^ mice. Of these, 1391 were up-regulated and 1988 were down-regulated (Table S1), indicating that the expression levels of large number of genes in MOE were altered in the absence of *AC3*. A complete array data is provided in the supplemental files.

### 2.2. Functional Analysis of Differentially Expressed Genes

**Figure 1:**
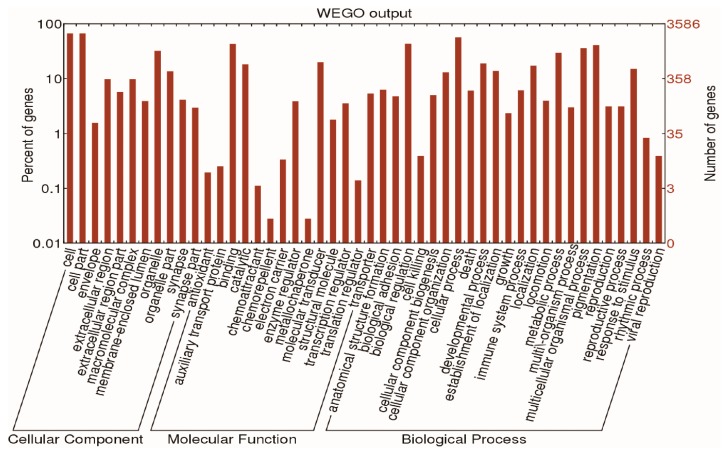
Web Gene Ontology Annotation Plot (WEGO) analysis of the differential expression genes. The impact of *AC3* deletion on the regulation of genes is associated with biological activities such as growth and development, metabolism, ion transport, energy conversion *etc.* in the MOE.

Currently, Gene Ontology (GO) functional annotation and pathway analysis is the most common method used for the analysis of differentially expressed genes. From the GO annotation entries, the differentially expressed genes regulated by *AC3* were involved in the regulation of biological processes including cell growth, apoptosis, metabolism, ion transportation, enzyme activity regulation, and positive and negative pressure stability of the neuronal cells (Figure 1). Pathway analysis revealed that after inactivating the *AC3* gene, the differentially expressed genes were involved in many signaling pathways including fat metabolism, cell growth and apoptosis, as well as tissue development *etc.* (Table 1).

**Table 1:** Pathway analysis of the differentially expressed genes by using the KEGG database.

Pathway Name	*p*-Value	Hit	Total	Percent
Metabolism correlation				
Alanine aspartate and glutamate metabolism *	0.0101	6	34	17.65%
alpha-Linolenic acid metabolism	0.132	4	20	20%
Amino sugar and nucleotide sugar metabolism *	0.046	5	49	10.2%
Arachidonic acid metabolism	0.081	12	56	21.43%
Arginine and proline metabolism **	0.0096	5	26	19.23%
Ascorbate and aldarate metabolism	0.109	5	26	19.23%
Fatty acid metabolism *	0.046	20	50	40%
Histidine metabolism	0.134	5	28	17.86%
Inositol phosphate metabolism *	0.027	17	57	29.8%
Fat digestion and absorption *	0.024	16	45	35%
beta-Alanine metabolism **	0.0082	8	25	29%
Linoleic acid metabolism *	0.023	8	46	17.39%
Disease association				
Type I diabetes mellitus **	0.000	21	63	33.3%
Olfactory transduction **	0.0	395	1041	35%
Graft-versus-host disease **	0.0	19	58	32.76%
Parkinson’s disease	0.9	9	151	5.98%
Chagas disease *	0.0324	16	101	15.84%
Alzheimer’s disease *	0.037	19	75	25.3%
Autoimmune thyroid disease **	0.0004	18	72	25%
Allograft rejection **	0.0001	17	56	33.36%
Arrhythmogenic right ventricular cardiomyopathy (ARVC) **	0.0	22	74	29.73%
Dilated cardiomyopathy **	0.0	24	89	26.97%
Hypertrophic cardiomyopathy (HCM) **	0.0	24	83	28.92%
Cell growth and death				
Natural killer cell mediated cytotoxicity **	0.005	22	125	17.6%
Mitogen-activated protein kinase (MAPK) signaling pathway *	0.0136	37	296	13.75%
Ras-Independent pathway in NK cell-mediated cytotoxicity *	0.0313	5	17	29.41%
p53 signaling pathway *	0.038	18	70	25.7%
Sexual maturity				
Maturity onset diabetes of the young *	0.0453	6	26	23.08%
Oocyte meiosis *	0.043	17	114	14.91%
GnRH signaling pathway *	0.024	23	99	23.23%
Metabolism of xenobiotics by cytochrome P450 *	0.011	11	78	15.1%
Neural development				
IL12 and Stat4 Dependent Signaling Pathway in Th1 Development **	0.0082	7	22	31.82%
Th1/Th2 Differentiation **	0.0034	7	18	38.89%
Neurotrophin signaling pathway *	0.043	17	50	34%
Osteoclast differentiation *	0.032	13	75	17.3%

Hit represents the numbers of differentially expressed genes identified by microarray hybridization on the given term. Total represents the total gene numbers on the given term. Percent means the % of hit to total. * means *p* < 0.05 representing a significant difference; and ** means *p* < 0.01 representing a highly significant difference.

### 2.3. Effect of AC3 Knockout on cAMP Signaling Pathway-Related Genes

The cAMP signal transduction pathway is the main pathway in animals for the detection of odor molecules. In the cAMP olfactory signal transduction pathway, the role of *AC3* is to receive stimulation from the Golf protein and to carry out the conversion of ATP to cAMP. KEGG data shows that 1014 genes are involved in the cAMP olfactory signal transduction pathway. Our microarray hybridization showed that 395 genes were differentially expressed between the *AC3*^−/−^ and *AC3*^+/+^ mice, including key genes in the cAMP olfactory signal transduction pathway including olfactory receptors (ORs), Golf and CNG *etc.* The ORs account for the highest number of differentially expressed genes. There are over 1000 ORs in mice [2], distributed on all chromosomes except the Y chromosome. A total of 1060 ORs were detected in our microarray hybridization. Among them, the expression levels of 425 were significantly different between the *AC*3^−/−^ and *AC*3^+/+^ mice, representing 40% of all the ORs (Table S2). In these 425 ORs, 28 belongs to class I subfamily, 397 belongs to class II subfamily (Table S2). Of these 425 ORs, 97.88% were down-regulated, while only 2.12% of them were up-regulated.

### 2.4. Effect of AC3 Knockout on the Expression of Genes Related to Cell Growth and Apoptosis, and on Their Signal Pathways

**Figure 2:**
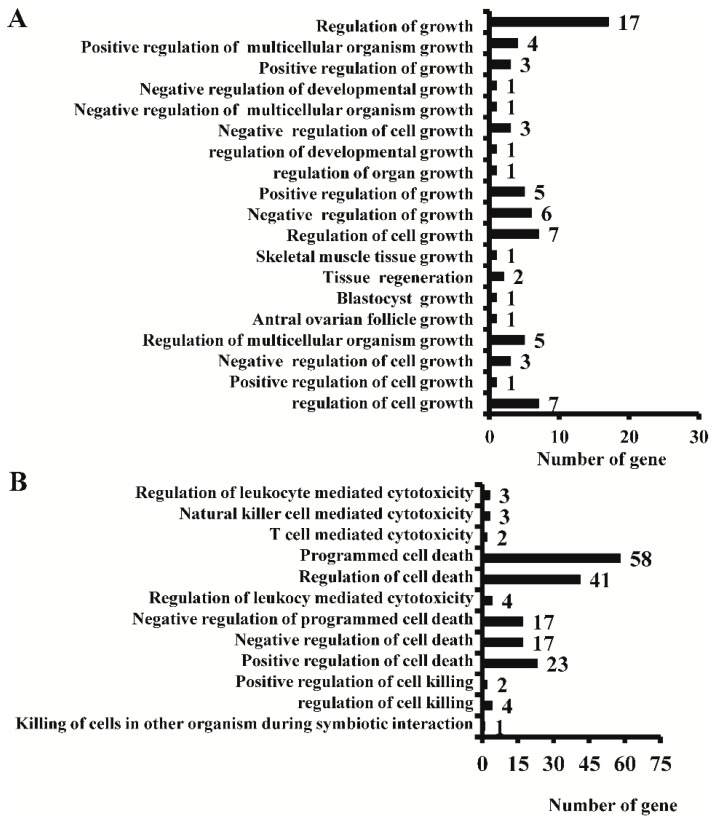
GO annotation results on the effect of *AC3* knockout on cell growth and death related function genes. (**A**) GO annotation results on the effects of *AC3* knockout on cell growth-related function genes; (**B**) GO annotation results on the effects of *AC3* knockout on cell death-related function genes.

The GO annotation analysis showed that many differentially expressed genes identified in *AC*3^−/−^ mice were associated with cell growth and mitosis (Figure 2A,B). Calcium/calmodulin-dependent protein kinase II (*CaMKII*) is a key gene in the calcium ion signaling pathway and it also plays a role in other important physiological functions, including cell morphology maintenance [29], apoptosis [30,31] and cell migration [32]. In the *AC3* deficient mouse MOE, many key genes associated with the calcium ion signaling pathways were differentially expressed, including *CaMKII* (Figure S1A,B). Mitogen-activated protein kinases (MAPKs) are a group of serine-threonine protein kinases widely expressed in eukaryotic cells. Members of its family include the extracellular signal-regulated kinase (ERK), c-Jun N-terminal kinase (JNK) and p38-MAPK. After the activation of p38-MAPK, the expression of genes promoting apoptosis such as transcription factor *ATF-2*, *Elk-1* and *CHOP10* are activated by a cascading chain reaction. This process plays an important role in apoptosis [33]. When *AC3* was deleted, the expression of the genes related to this MAPK signal transduction pathway were altered (Figure S2A,B). The Notch/Delta signaling pathway plays a regulatory role during embryonic development. There are four recognized key members of the Notch signaling pathway, namely Notch1, presenilin 1 (PS1), recombing binding protein suppressor of hairless (RBP-Jk) and HES1/5. In the MOE of *AC3*^−/−^ mice, the expression of *Notch1* and *HES1/5* was significantly altered (Figure S3A,B). Furthermore, the Wnt signaling pathway plays an important role in the process of the embryonic neurogenesis. Using RT-PCR, Lee *et al.* [34] reported that the expression of *wnt3a*, *wnt4*, *wnt5a*, *wnt7a* and *wnt2b* were detected from the cerebral tissues of mouse embryos during embryonic period E12.5. Among these *Wnt* genes, *wnt1*, *wnt3a* and *wnt5a* play important roles in neurogenesis, and promote neural stem cell proliferation [35]. In the MOE of *AC3*^−/−^ mice, the expression of a large number of genes associated with the Wnt signaling pathway were altered (Figure S4A,B). In addition, after inactivation of *AC3*, the expression of genes associated with apoptosis, cell growth and development signaling pathways were also affected (Table 2, Figures S5 and S6). This shows that by inactivating the *AC3* gene, the expression of many genes involved in cell growth and apoptosis in MOE were altered and the functions of these signal transduction pathways were affected.

**Table 2:** Effect of *AC3* knockout on cell death related signaling pathways.

Pathway Name	Hits	Total	*p*-Value	Percent
Apoptotic DNA fragmentation and tissue homeostasis	1	9	0.000	11.1%
Apoptotic Signaling in Response to DNA Damage	3	21	0.0	14.2%
Erk and PI-3 Kinase Are Necessary for Collagen Binding in Corneal Epithelia	3	23	0.0	13%
Erk1Erk2 Mapk Signaling pathway	4	28	0.9	14.2%
Erythropoietin mediated neuroprotection through NF-kB	3	11	0.0324	27.3%
FAS signaling pathway (CD95)	3	28	0.037	10.7%
MAPKinase Signaling Pathway	5	77	0.0004	6%
p38 MAPK Signaling Pathway	4	31	0.0001	13%
p53 Signaling Pathway	4	16	0.0	25%
Th1/Th2 Differentiation	7	18	0.0	39%
WNT Signaling Pathway	2	25	0.0	8%

Hit represents the numbers of differentially expressed genes identified by microarray hybridization on the given term. Total represents the total gene numbers on the given term. Percent means the % of hit to total.

### 2.5. Effect of AC3 Knockout on the Expression of Genes Related to Olfactory Neuron Functions

**Figure 3:**
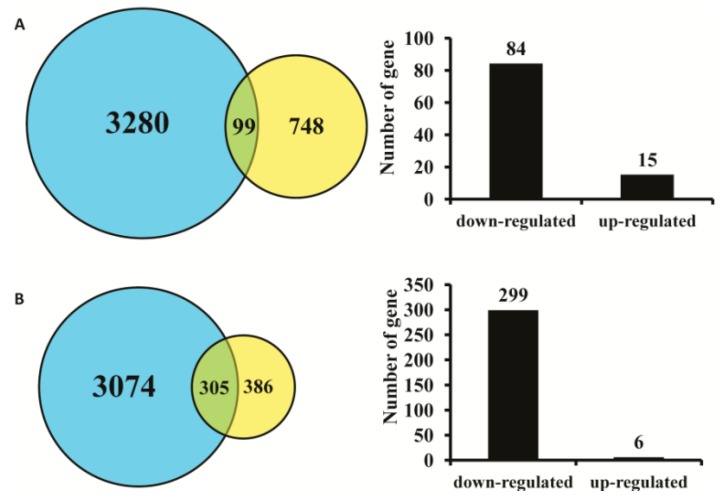
The impact of *AC3* deletion on the expression of mature and immature olfactory neuron-specific genes. (**A**) Comparison of the microarray results with the immature olfactory neuron-specific expression genes [21], 99 genes overlap with the microarray results, among them 15 and 84 genes were up-regulated and down-regulated, respectively; (**B**) Compared results between the microarray data and the mature olfactory neuron-specific expression genes [21] indicating that 305 genes were overlapped, with 6 genes up-regulated and 299 genes down-regulated. In the Venn diagrams, the microarray results and the genes identified in [21] were marked in blue and yellow color, respectively.

Nickell *et al.* [21] found that 847 genes were specifically expressed in the immature olfactory neurons in mice, while 691 genes were specifically expressed in the mature olfactory neurons. Of the 847 genes specifically expressed in the immature olfactory neurons identified by Nickell *et al.*, 99 were found to be differentially expressed in our microarray analysis, with 15 being up-regulated and 84 down-regulated. Among the 691 genes specifically expressed in the mature olfactory neurons (identified by Nickell *et al.* [21]), 305 were differentially expressed in our microarray results, of which six were up-regulated and 299 were down-regulated (Figure 3A,B, Tables S3 and S4). The expression levels of a large number of genes specifically expressed in the immature and mature olfactory neurons were altered in the absence of *AC3*, indicating that *AC3* plays an important role in the regulation of physiological functions of the olfactory neurons.

### 2.6. Effect of AC3 Knockout on Epigenetic Regulation Genes

Epigenetics refers to acquired genetic changes of gene function or gene expression that are not involved with DNA sequence variation. The microarray hybridization results revealed that *AC3* inactivation affected the expression of many epigenetic regulation genes related to methylation, histone modification, RNA silencing and genetic imprinting (Table S5).

### 2.7. qRT-PCR Validation of the Differentially Expressed Genes

**Figure 4:**
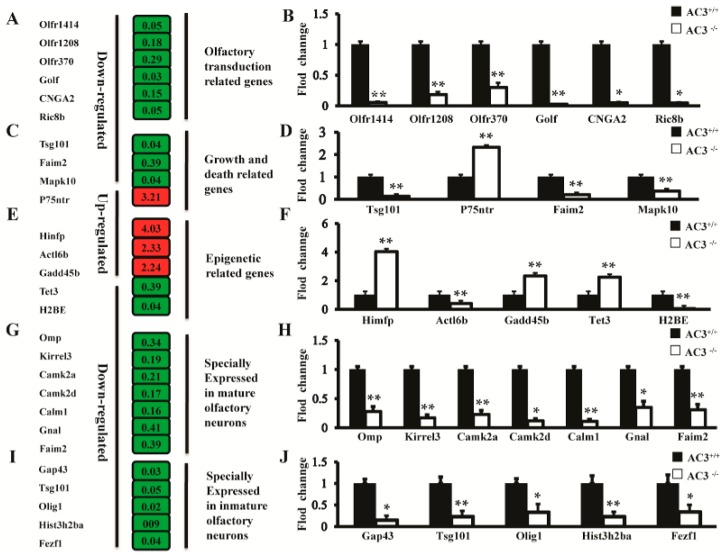
Quantitative RT-PCR verification of the differentially expressed genes in the AC3^−/−^ and AC3^+/+^ mice. (**A**) Differentially expressed genes associated with olfactory signal transduction function identified from the microarray results; (**B**) qRT-PCR verification of the differentially expressed genes associated with olfactory signal transduction; (**C**) Differentially expressed genes associated with cell growth and death identified from the microarray results; (**D**) qRT-PCR verification of the differentially expressed genes associated with cell growth and death; (**E**) Differentially expressed genes associated with epigenetic regulation function identified from the microarray results; (**F**) qRT-PCR verification of the differentially expressed genes associated with epigenetic regulation functions; (**G**) Differentially expressed genes specifically expressed in the mature olfactory neurons identified from the microarray results; (**H**) qRT-PCR verification of the differentially expressed genes specifically expressed in mature olfactory neurons; (**I**) Differentially expressed genes specifically expressed in the immature olfactory neurons identified from the microarray results; (**J**) qRT-PCR verification of the differentially expressed genes specifically expressed in immature olfactory neurons. Note: In the microarray result panel, green represents down-regulated genes and red represents up-regulated genes. The numbers in the red and green boxes represent fold change. *n* = 3 for each group of mice, experiments are in quadruplicates. All values are expressed as mean ± s.d. * *p* ≤ 0.05, ** *p* ≤ 0.01, ANOVA with Student’s *t*-test.

Based on the fold changes of expression level between *AC3*^−/−^ and *AC3*^+/+^ mice, as well as their physiological functions, 38 differentially expressed genes were selected for qRT-PCR verification. The results showed that five genes were indistinguishable at expression levels between *AC3*^−/−^ and *AC3*^+/+^ mice, three genes had no PCR products produced, and three genes were inconsistent with the microarray hybridization. Whereas qRT-PCR results of 27 differentially expressed genes were consist with the microarray hybridization. The qRT-PCR results from the cAMP olfactory signaling pathway associated genes, including *CNGA2*, *Golf*, *Ric8b*, *Olfr370*, *Olfr1414*, *Olfr1208* and *Ric8b* were significantly down-regulated (Figure 4A,B). As for the cell growth and death associated signaling pathways, qRT-PCR results from *P75NTR*, *Faim2*, *Tsg101* and *Mapk10* showed that the expression of *Faim2*, *Tsg101* and *Mapk10* were significantly down-regulated, while the expression of *p75NTR* was significantly up-regulated (Figure 4C,D). As for the epigenetic regulation related genes, qRT-PCR was also conducted for validation of the microarray data from the genes that play important roles in DNA methylation and protein modification, such as *Hinfp*, *Actl6b*, *Tet3*, *Gadd45* and *H2BE*. The results showed that *Hinfp*, *Tet3* and *Gadd45* were significantly up-regulated after *AC3* inactivation, while *Actl6b* and *H2BE* were significantly down-regulated (Figure 4E,F). In terms of neurogenesis, 25 genes that play important roles in the neurogenesis of the MOE were identified by Wang *et al.* [13], of which, *Omp*, *Kirrel3*, *Camk2a*, *Camk2d*, *Calm1*, *Gnal* and *Faim2* were specifically expressed in the mature olfactory neurons (Figure 4G,H) and *Gap43*, *Tsg101*, *Olig1*, *Hist3h2ba* and *Fezf1* were specifically expressed in the immature olfactory neurons (Figure 4I,J). The qRT-PCR results showed that similar changes as microarray hybridization have taken place in the expression of these genes in *AC3*^−/−^ and *AC3*^+/+^ mice (*p* < 0.01).

## 3. Discussion

Olfactory sensation plays an important role in mammalian life. The occurrence of many diseases is accompanied by loss of olfaction and olfactory memory loss, including Alzheimer’s disease (AD) and Parkinson disease (PD) [36,37]. Olfactory detection of odor molecules in mice depends primarily on the cAMP olfactory signal transduction pathway in the MOE [38]. *AC3* is a component of the cAMP olfactory signal transduction pathway. Loss of *AC3* function not only results in olfactory dysfunction in mice, but also leads to behavioral disorders, as well as abnormal IP3 signal transduction in the MOE [39]. However, it has yet to be determined whether *AC3* gene deletion in the murine MOE will affect the expression of genes related to networking groups. In this study, using *AC3* knockout mice and wild-type mice as a comparison, differentially expressed genes in the MOE between *AC3*^−/−^ and *AC3*^+/+^ mice were identified using microarrays. It was found that altered expressions of genes included not only those related to olfactory function, but also those involved in other physiological functions, such as ion transport, metabolism, neurological development and epigenetic regulation, as well as those that regulate corresponding signal transduction pathways, such as substrate binding, cell cycle, biological and cellular processes. These data indicated that when *AC3* is lost, global gene expression and protein function within the mouse MOE are affected.

Our microarray hybridization results showed that, compared to *AC3*^+/+^ mice, the expression of many genes associated with the cAMP olfactory signal transduction pathway in the MOE of *AC3*^−/−^ mice was significantly altered. There were 1014 genes detected in the mouse cAMP olfactory signal transduction pathway, of which the expression levels of 395 genes were altered, including OR, Golf and the CNG channel. The combination of odor molecules with ORs in the cAMP signal transduction pathway is the first step in the detection of smells. The type of OR and its expression level are important factors that influence olfaction. Mammalian olfactory acuity and the number of functional ORs in the genome are positively correlated [40,41,42]. The number of ORs may reflect the ability of the animal to smell [43]. Compared with other primates, the low number of olfactory receptor genes in humans is an important cause of olfactory deterioration [44,45]. Our microarray results showed that, when the *AC3* gene was deleted, expression of 425 OR genes was significantly altered in the MOE, representing 40% of the total 1060 ORs detected (Table S2), of which 97.88% were down-regulated, while only 2.12% were up-regulated. In addition, of the genes specifically expressed in immature olfactory neurons [21], 99 were found to be differentially expressed in our microarray analysis, with 15 being up-regulated and 84 being down-regulated. Among the 691 genes specifically expressed in mature olfactory neurons [21], 305 were differentially expressed in our microarray results, of which six were up-regulated and 299 were down-regulated (Figure 3A,B, Tables S3 and S4). In summary, the expression of many genes related to the cAMP olfactory signal transduction pathway, ORs, and olfactory receptor neuron (ORN) development was significantly down-regulated in the MOE of *AC3*^−/−^ mice. These might be caused by the reduction of cell number, and thickness of the neuronal layer in the MOE of *AC3*^−/−^ mice as revealed in many olfactory related gene knockout mice. For example, a decrease of 20% was revealed in both thickness and cell number in the MOE of NKCC1-deficient mice [46]. In the ATF5 knockout (KO) MOEs, the numbers of OR-expressing neurons are significantly reduced [15]. Reduced ORs cell densities and thin MOE with increased apoptotic cells were demonstrated in OMP-Kir2.1 mice [47]. The number of mature ORNs was reduced 30% in OAZ knockout mice [48]. It was originally reported that the *AC3*^−/−^ mice has a grossly normal MOE structure [4]. However, our microarray investigation demonstrated that the expressing levels of OMP and Golf was significantly downregulated, indicating that the ORN cell number might be reduced in the MOE of *AC3*^−/−^ mice.

Activity-dependent survival of ORNs may allow animals to tune their olfactory systems to match their odor environment. By using CNGA2 knockout mice and microarray analysis, Bennett *et al.* found that activity-regulated expression of endogenous genes were highly regulated by odorant environments [27]. Microarray analysis with unilateral naris occlusion (UNO), a popular mouse model of stimulus deprivation, showed that compensatory responses of gene transcription could be induced in both occluded and non-occluded sides of MOE [25]. In the present study, 3379 genes were altered with greater than 2.0-fold at expression levels in *AC3*^−/−^ mice, with 1391 were up-regulated and 1988 were down-regulated (Table S1). Since ORNs of the MOE in the *AC3*^−/−^ mice are essentially devoid of odorant-evoked activity, the odor environment cues may contribute to altered expressions of genes in the *AC3*^−/−^ mice. The up-regulated alteration in the *AC3*^−/−^ mice might be caused by compensatory response to the odor environment interpreted as homeostatic among different cell types within the MOE [25].

Although *AC3*^−/−^ mice are devoid of odor environment cues, in the present study only 40% of ORs were differentially regulated by the deletion of *AC3*. Since *AC2* and *AC4* are expressed in olfactory cilia of *AC3*^−/−^ mice [4], expression of other adenylyl cyclases may eventually generate enough cAMP to maintain the expression of 60% of the ORs. In addition, other cell types without expressing *AC3* [49], or other signal pathways [50,51] within the MOE may also contribute to the expression of ORs that is not regulated by *AC3 in the AC3*^−/−^ mice. Moreover, in the present microarray study, nine ORs, about 2% of the total regulated ORs, were up-regulated in the *AC3*^−/−^ mice. Up-regulated ORs in the *AC3*^−/−^ mice might be indispensable for suckling and be evoked by odorant cues for compensatory responses, because different ORs might be differentially stimulated in postnatal development [47].

An advantage of RNAseq over the other expression profiling techniques is that it is not restricted to a catalog of known transcripts. Recently, RNA profiles from MOEs of both male and female mice, as well as from fluorescence-activated cell sorting (FACS)-sorted olfactory receptor neurons, was revealed [52,53,54,55]. A comprehensive list of transcripts in the MOE, and expression profiles between ORNs and other cell types in the MOE were provided. In order to obtain information of specific genes expression on ORN and of genes expressed in other cell types regulated by *AC3 in the* MOE, in future, deep sequencing of RNAs in combination with FACS-sorted ORNs, as well as single-cell RNA sequencing [56], should be investigated in the *AC3*^−/−^ and *AC3*^+/+^ mice.

## 4. Experimental Section

### 4.1. Animals and Sample Preparation

*AC3^+/−^* mice with C57BL/6J background were from Storm’s laboratory, University of Washington, Seattle, United States. The mice were bred in the Specific Pathogen Free (SPF) animal rooms of Hebei University. The offspring from *AC3^+/−^* × *AC3^+/−^* breeding were genotyped using PCR according to previous reports [57]. *AC3^−/−^* and *AC3^+/+^* litters were sacrificed by cervical dislocation, and the MOE were dissected and immediately placed in RNA Later (Sigma, San Francisco, CA, USA). After storage at 4 °C overnight, the samples were placed at −80 °C. All operation procedures and processing methods used for the experimental animals were in line with the Guiding Opinions on the Treatment of Experimental Animals issued by the Ministry of Science and Technology, People’s Republic of China and approved by the Animal Ethics and Caring Committee of Hebei University.

### 4.2. Total RNA Extraction and cDNA Synthesis

Total RNA was extracted using TRIzol (Invitrogen, Carlsbad, CA, USA) and the extracted RNA quality was determined with a NanoDrop (Thermo, Waltham, MA, USA). Quality was also determined by electrophoresis, and then reverse transcribed into cDNA by using the Prime Script™ RT Reagent Kit with gDNA Eraser (TaKaRa, Dalian, China) for qRT-PCR.

### 4.3. Microarray Hybridization and Screening of the Differentially Expressed Genes

The MOE from three-month-old *AC3^+/+^* or *AC3^−/−^* mouse was used as the representative sample of the group. The samples were subjected to microarray hybridization in Shanghai Bohao Biotech Co. (Shanghai, China).

After obtaining the raw data, preliminary processing was conducted. The data with tag A were deleted to ensure the credibility of initial fluorescence values. Subsequently, the Shbioship (SBC) online analysis system provided by Bohao Company was used (http://SAS.ebioservice.com:10080/portal/root/molnet_shbh/index.jsp). The fold value (the difference in fold) represents the degree of differential expression between two genotype mice. The standard used to judge differential expression was as follows: gene expression from the knockout group was used as the valid gene. Compared to the wild type group, fold change <1.0 was considered as a down-regulated gene, while fold change >1.0 was considered as an up-regulated gene. Genes with a fold change ≥2, or ≤0.5 compared to the wild-type group were selected for further analysis.

### 4.4. Functional Annotation of the Differentially Expressed Genes

GO is a collaborative project developed by the Gene Ontology Consortium (GOC), and GO has become an extremely important method and tool in the field of bioinformatics. Currently, 3 independent ontologies have been established by GOC database: the biological process, the molecular function and the cellular component. The GO annotations of the differentially expressed genes were analyzed primarily using the online analysis system provided by Shanghai Bohao Company for the three functional analyses, namely Gene to Term, Term to Gene and Term Pie Picture. The distribution ratios of the differentially expressed genes in the three ontologies were used to identify the effect of the particular genes in the biological processes, molecular functions, and in the cellular components.

### 4.5. The Pathway Analysis for Differentially Expressed Genes

KEGG (Kyoto encyclopedia of genes and genomes) is a genome annotation database that integrates information from genomics, chemical and system functions. The most striking feature of KEGG is the powerful graphics functions, which uses a graphical display rather than text to present metabolic pathways and relationships between the various pathways, which allows researchers to have an intuitive and comprehensive understanding of the signal pathways in their research. In this study, two functional analyses using the on-line system provided by Shanghai Bohao Company (gene to pathway and pathway to gene) were used to identify the differentially expressed genes, and to locate the specific signaling pathways where the genes may actively participate. Throughout the analysis, the proportion of the differentially expressed genes in the specific signaling pathways and *p* values to determine the impact of the specific gene function in the signaling pathway were used.

### 4.6. qRT-PCR

The MOE from three month old *AC3^−/−^* (*n* = 3) and *AC3^+/+^* (*n* = 3) were dissected and RNAs were extracted with TRIzol (Invitrogen, Carlsbad, CA, USA). After checking the quality, the RNA samples were reverse transcribed into cDNA using the TaKaRa reverse transcription kit. Quantitative RT-PCR was performed using SYBR dyes and high performance PCR enzymes (Transgen Biotech, Bingjing, China). β-actin was used as the reference gene. The 2^−ΔΔ*C*t^ method was used for the statistical analysis of the qRT-PCR amplification results [58], with *p* < 0.05 representing a significant difference, and *p* < 0.01 representing a highly significant difference.

## 5. Conclusions

In this study, using the MOE from the *AC3^−/−^* and *AC3^+/+^* mice, and by microarray hybridization selection and fluorescence quantitative PCR (qRT-PCR) verification, we have found that after *AC3* deletion, gene expression in the MOE, including the olfactory receptor genes, the immature and mature olfactory neuron-specific genes, the epigenetic regulation factors, and many other genes, were significantly altered. Through the comprehensive regulation of the expression of these gene groups on epigenetic regulation, ion transport, neural development and differentiation, lipid metabolism and membrane protein transportation, *AC3* may play an important role in the regulation of olfactory signal transduction and odor detection function in murine MOE.

## Supplementary Materials

Supplementary materials can be found at http://www.mdpi.com/1422-0067/16/12/26107/s1.
