# Supplementary material for: Gene Expression Profiles of Main Olfactory Epithelium in Adenylyl Cyclase 3 Knockout Mice
Source: Int J Mol Sci. 2015 Nov 30;16(12):28320–33. doi: 10.3390/ijms161226107 (PMC4691054; doi:10.3390/ijms161226107)
Supplement: Supplementary file 1 [file ijms-16-26107-s001.zip › ijms-97743-supplementary.pdf]

Supplementary Materials: Gene Expression Profiles of Main Olfactory Epithelium in Adenylyl Cyclase 3 Knockout Mice

Zhenshan Wang, Yanfen Zhou, Yingtao Luo, Jing Zhang, Yunpeng Zhai, Dong Yang, Zhe Zhang, Yongchao Li, Daniel R. Storm and Runlin Z. Ma

(a)

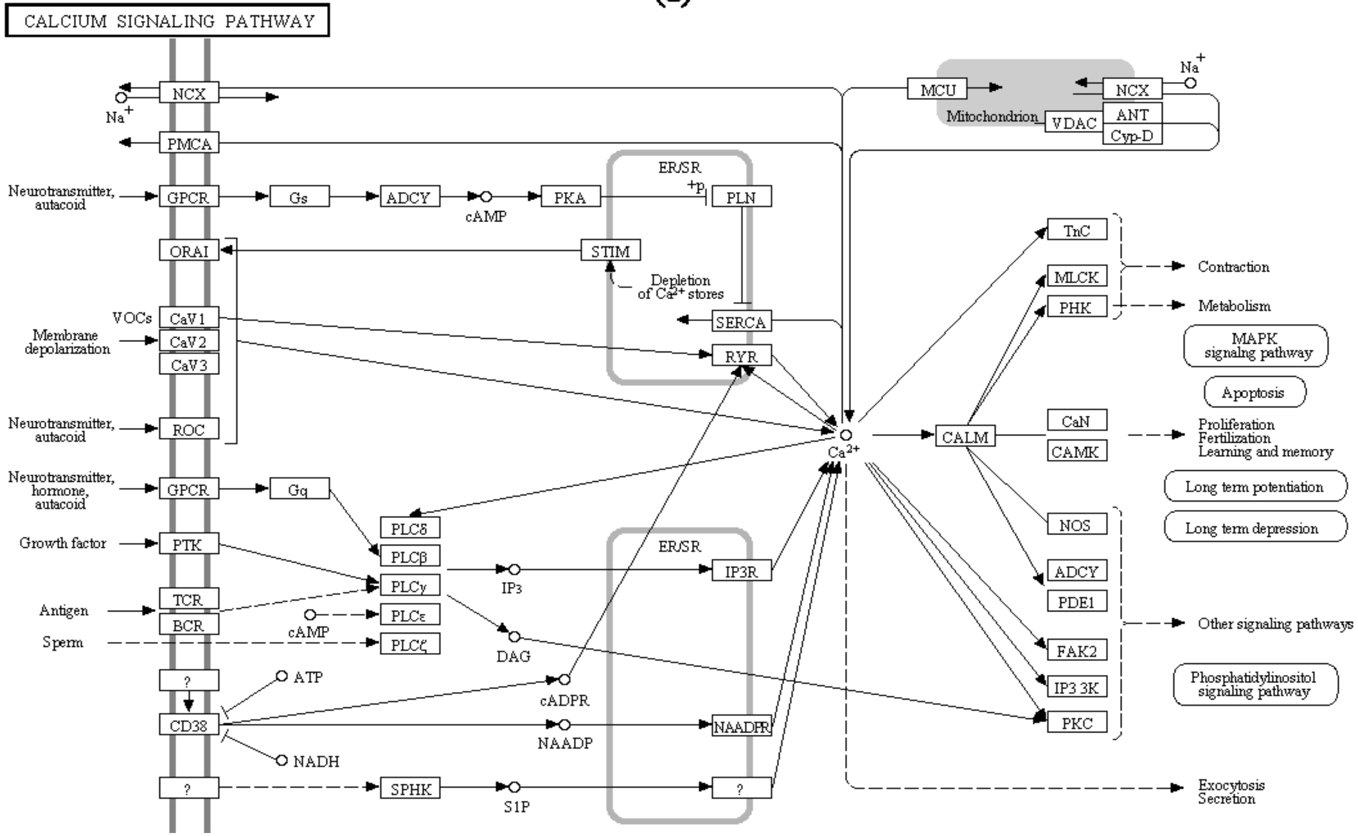

Figure S1. *Cont.*

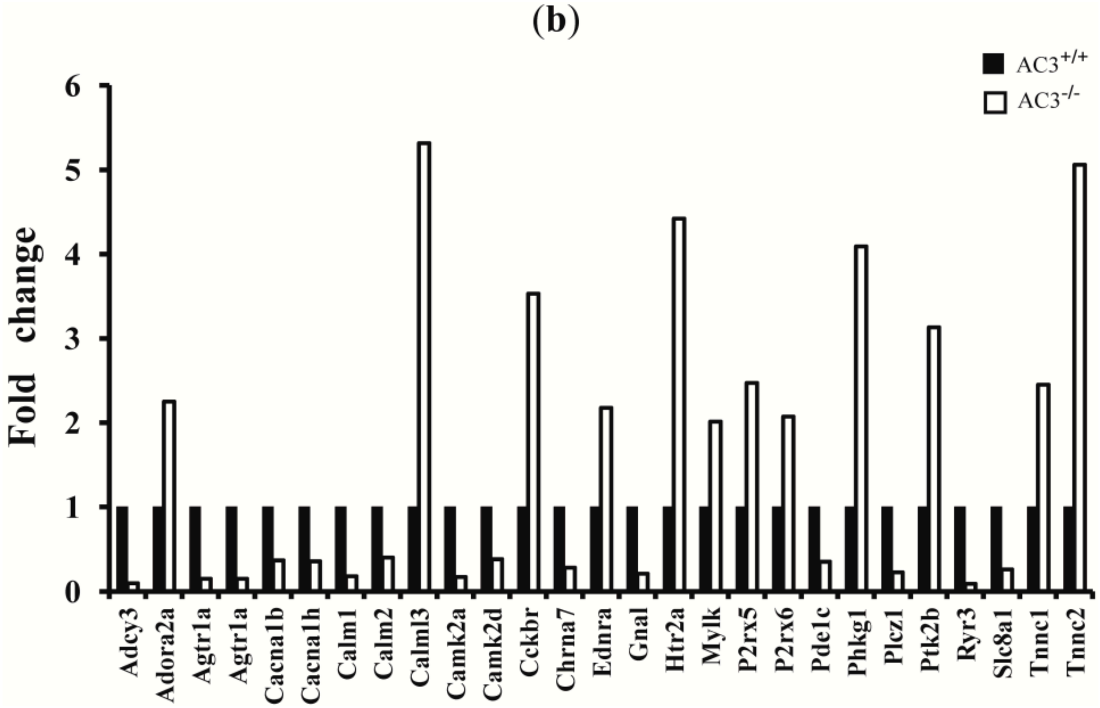

Figure S1. Expression level changes of genes involved in calcium ion signaling pathways after *AC3* deletion. (**a**) Influence of the differentially expressed genes on the calcium ion signaling and related signaling pathways; (**b**) The changes of the expression levels of the differentially expressed genes in the calcium ion signaling pathway from the microarray results.

(a)

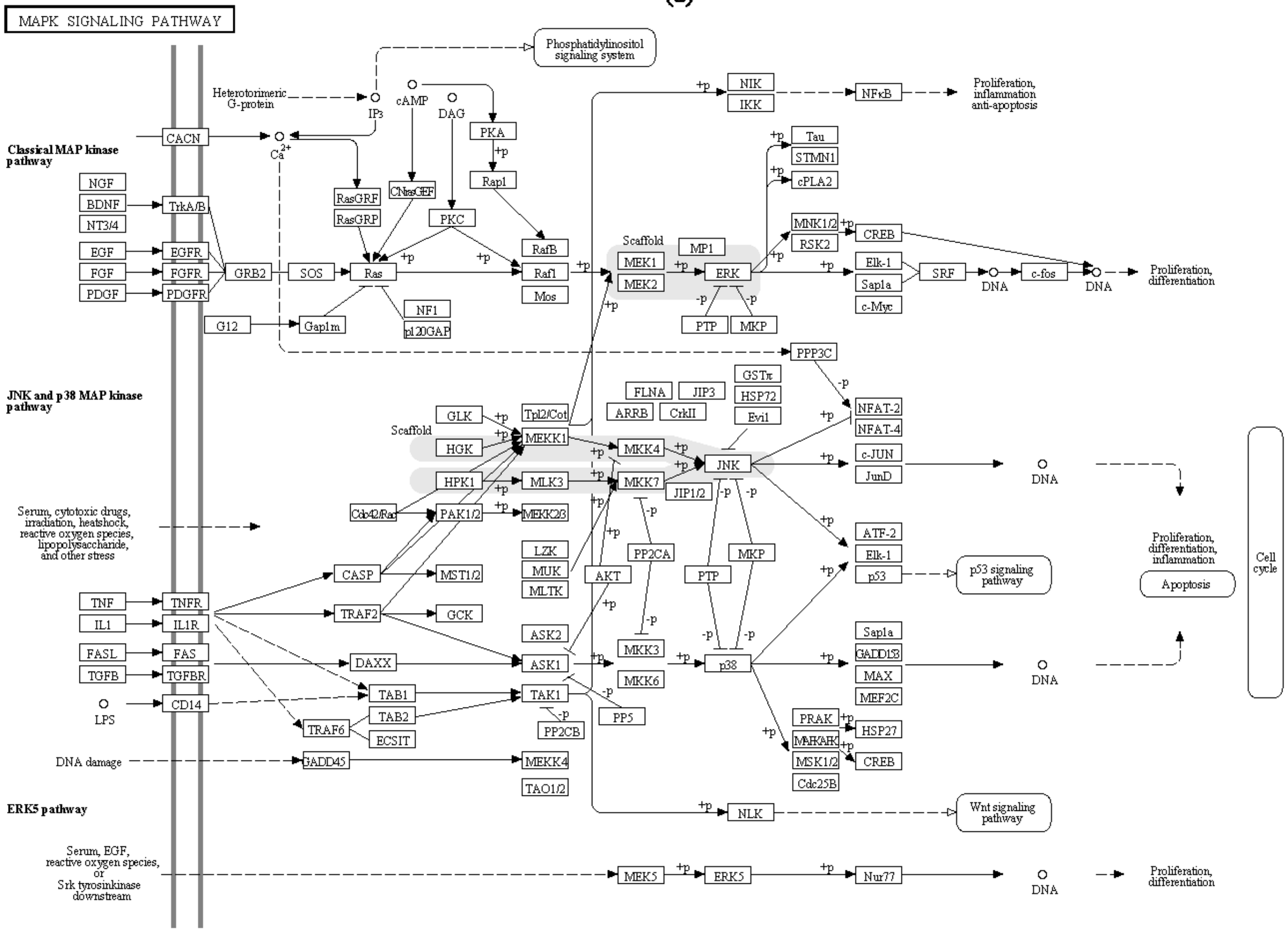

Figure S2. *Cont.*

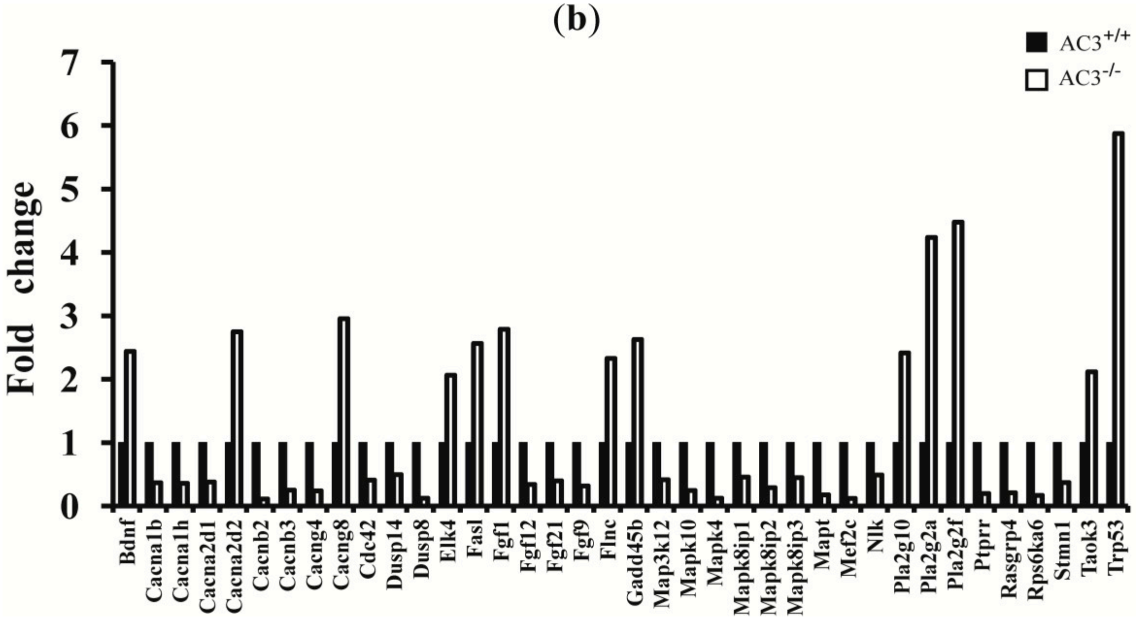

Figure S2. Expression level changes of the genes in the MAPK signal transduction pathway after *AC3* deletion. (**a**) Influence of the differentially expressed genes on MAPK signaling and related signaling pathways; (**b**) The changes of the expression levels of the differentially expressed genes in MAPK signaling pathway from the microarray results.

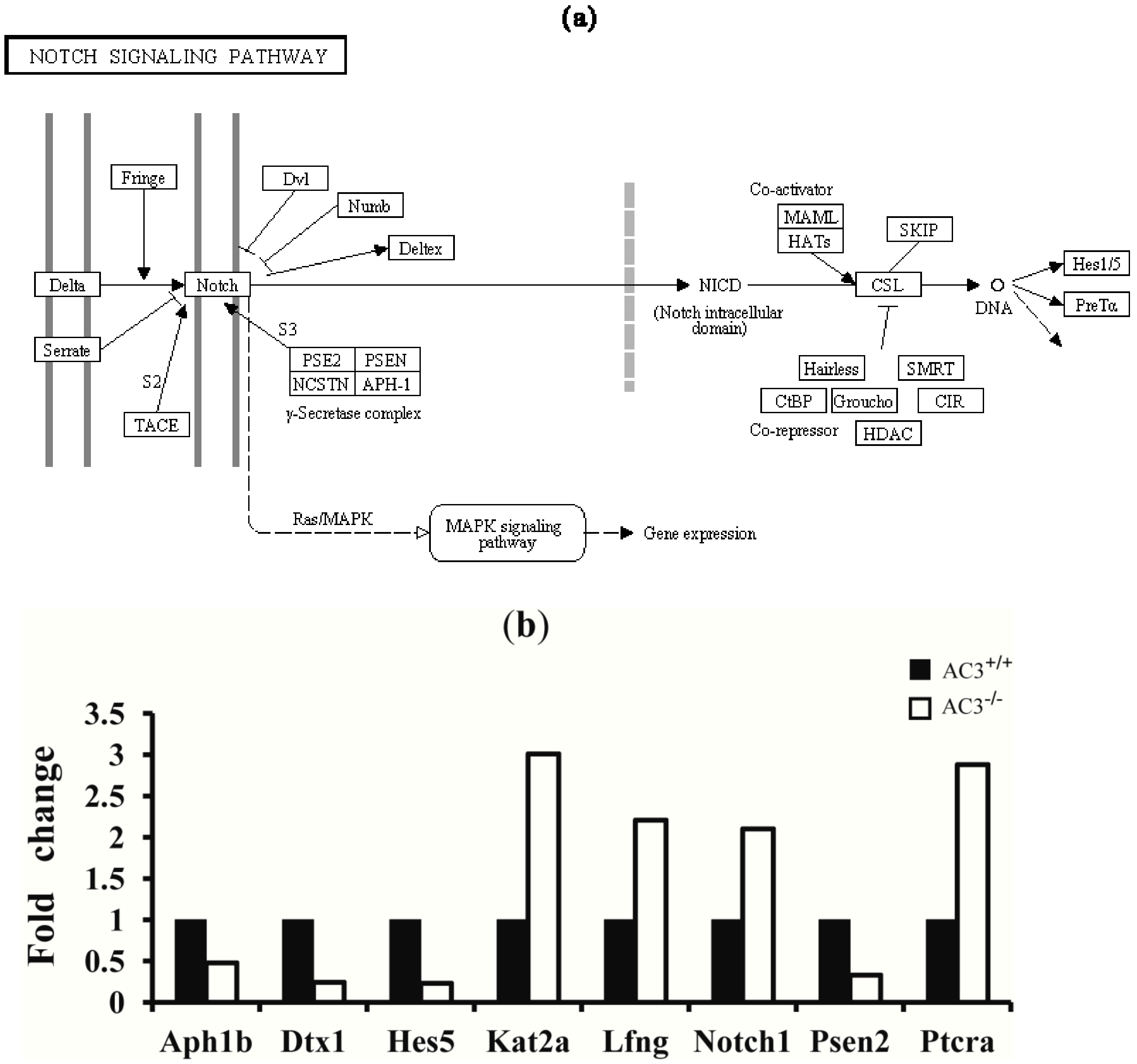

Figure S3. Expression levels of the genes in the Notch signal transduction pathway were altered after *AC3* deletion. (**a**) Influence of the differentially expressed genes on Notch signaling pathway and related signaling pathways; (**b**) The changes of the expression levels of the differentially expressed genes in Notch signaling pathway from the microarray results.

(a)

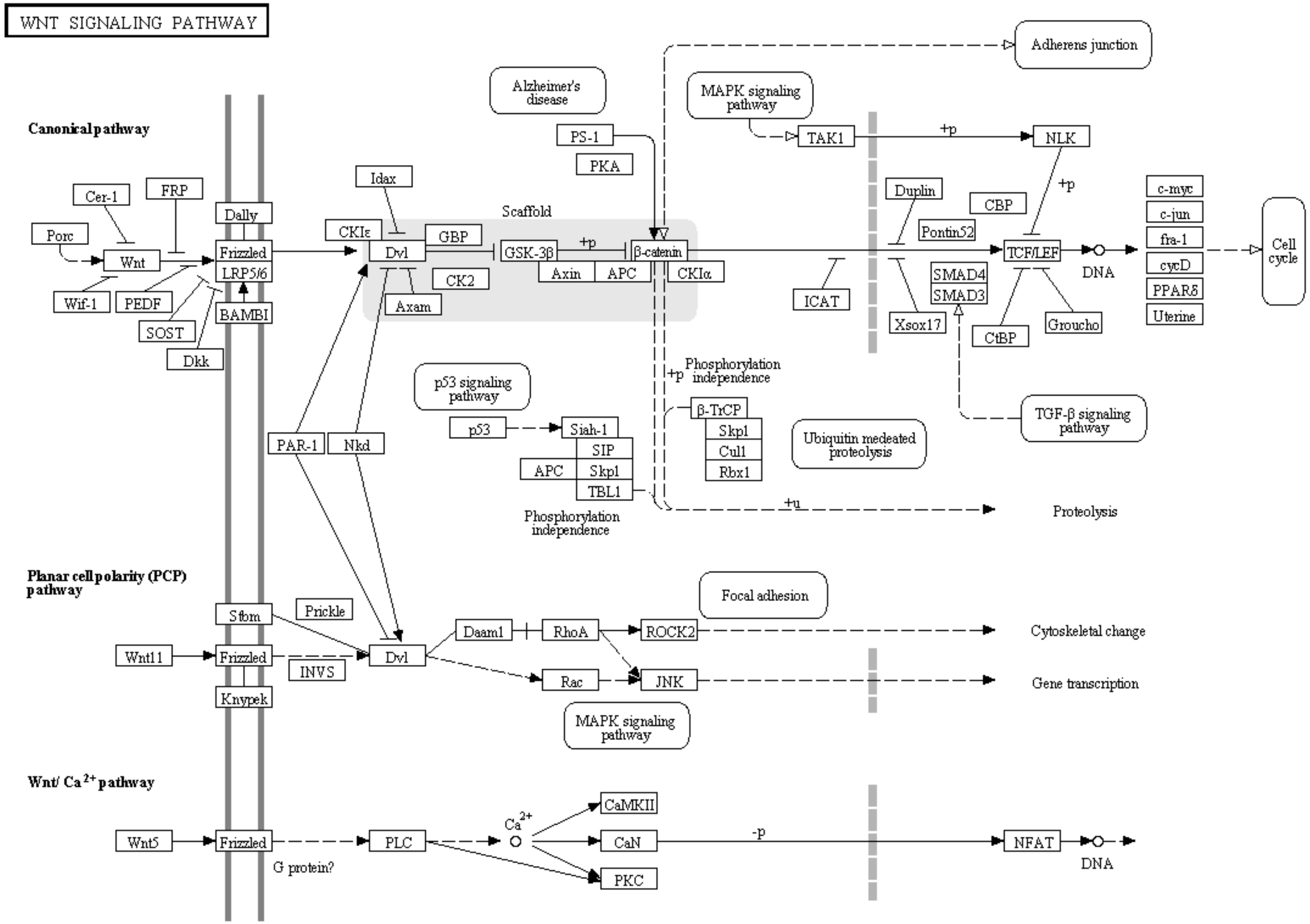

Figure S4. *Cont.*

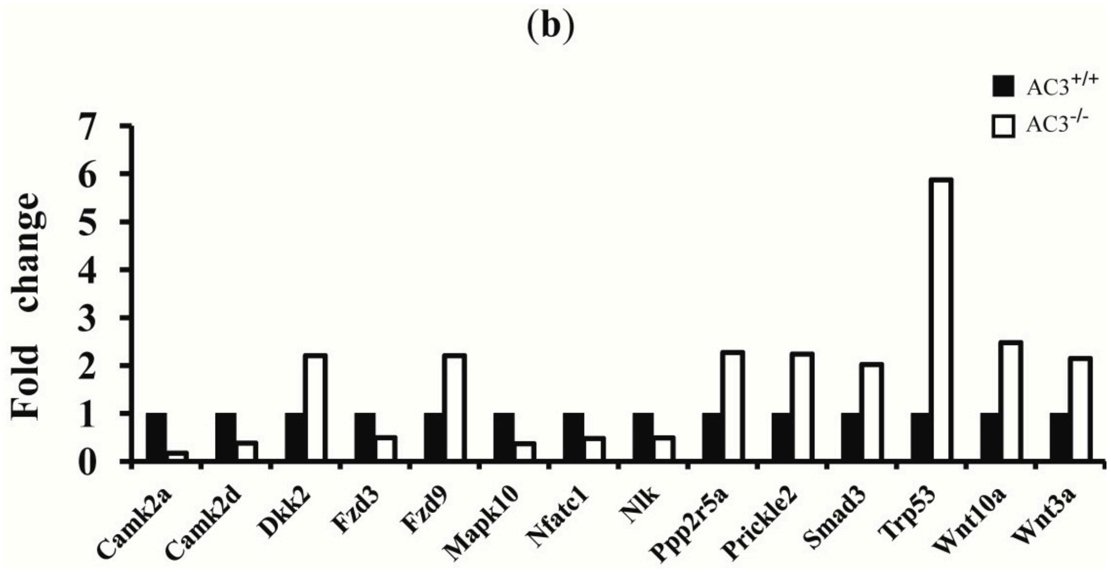

Figure S4. Expression levels of the genes in the Wnt signal transduction pathway were altered after *AC3* deletion. (**a**) Influence of the differentially expressed genes on Wnt signaling pathway and related signaling pathways; (**b**) The changes of the expression levels of the differentially expressed genes in Wnt signaling pathway from the microarray results.

(a)

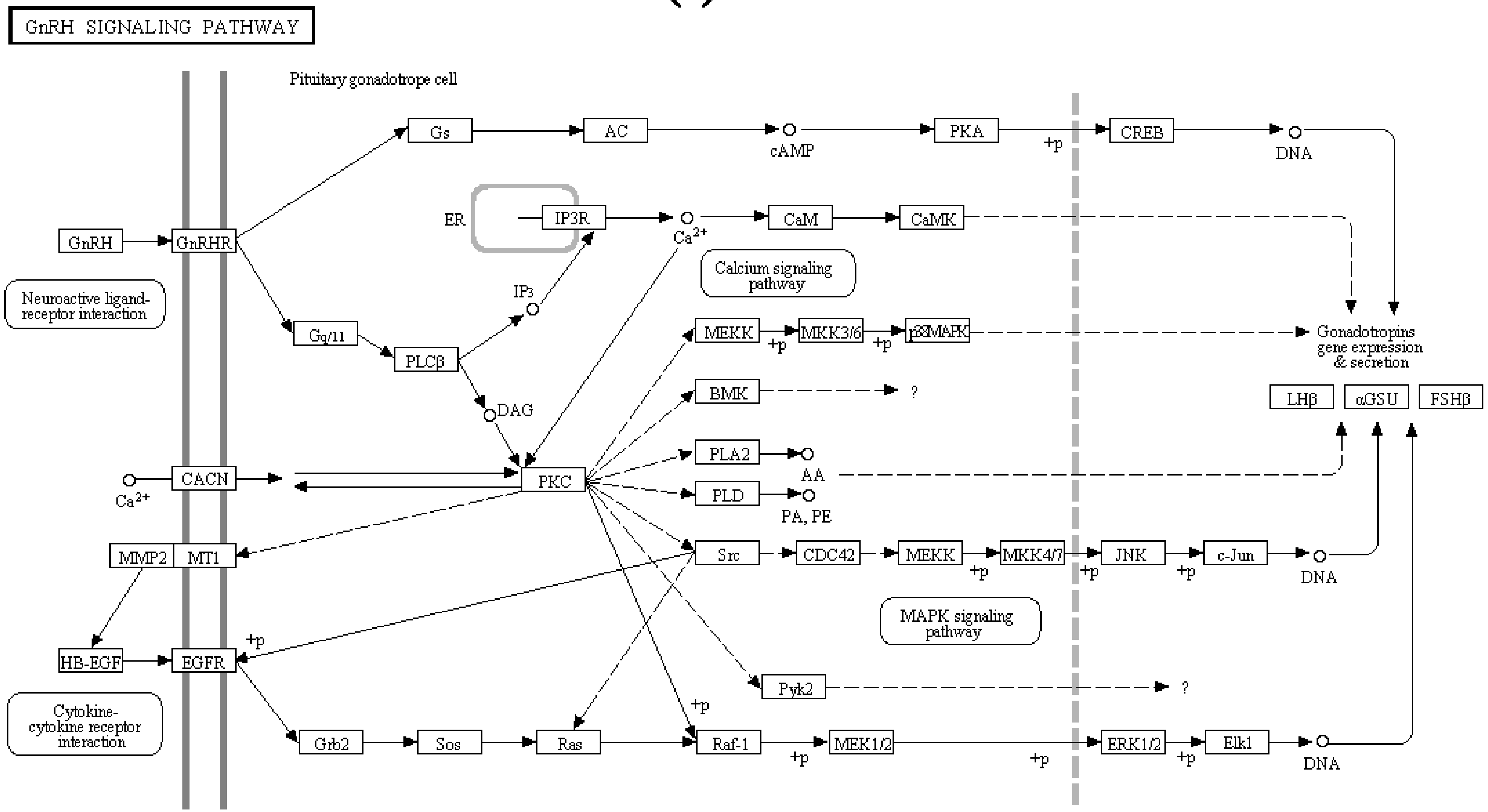

Figure S5. *Cont.*

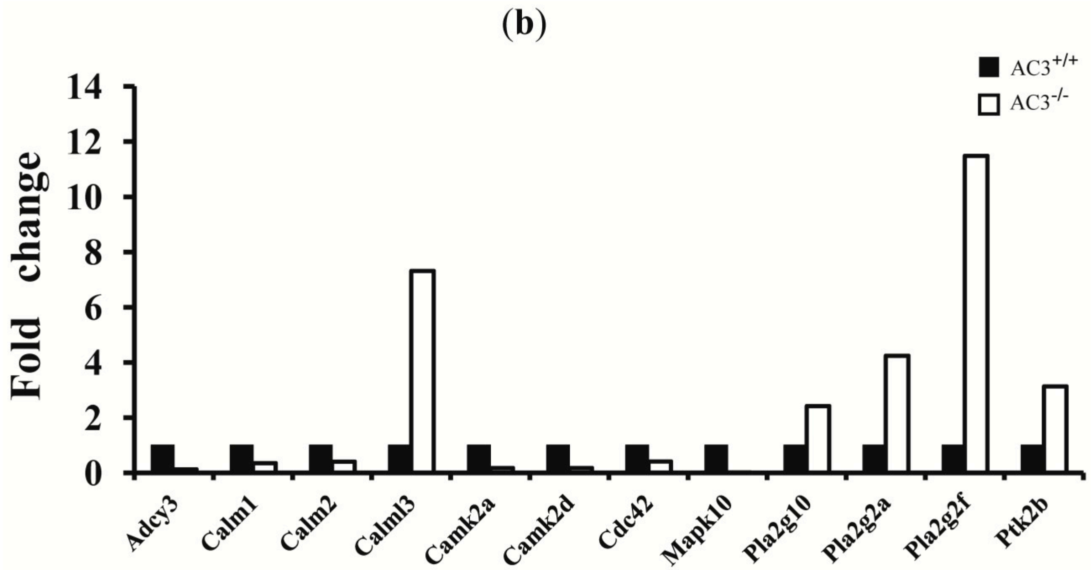

Figure S5. Expression levels of the genes in the GnRH signal transduction pathway were altered after *AC3* deletion. (**a**) Influence of the differentially expressed genes on GnRH signaling pathway and related signaling pathways; (**b**) The changes of the expression levels of the differentially expressed genes in GnRH signaling pathway from the microarray results.

(a)

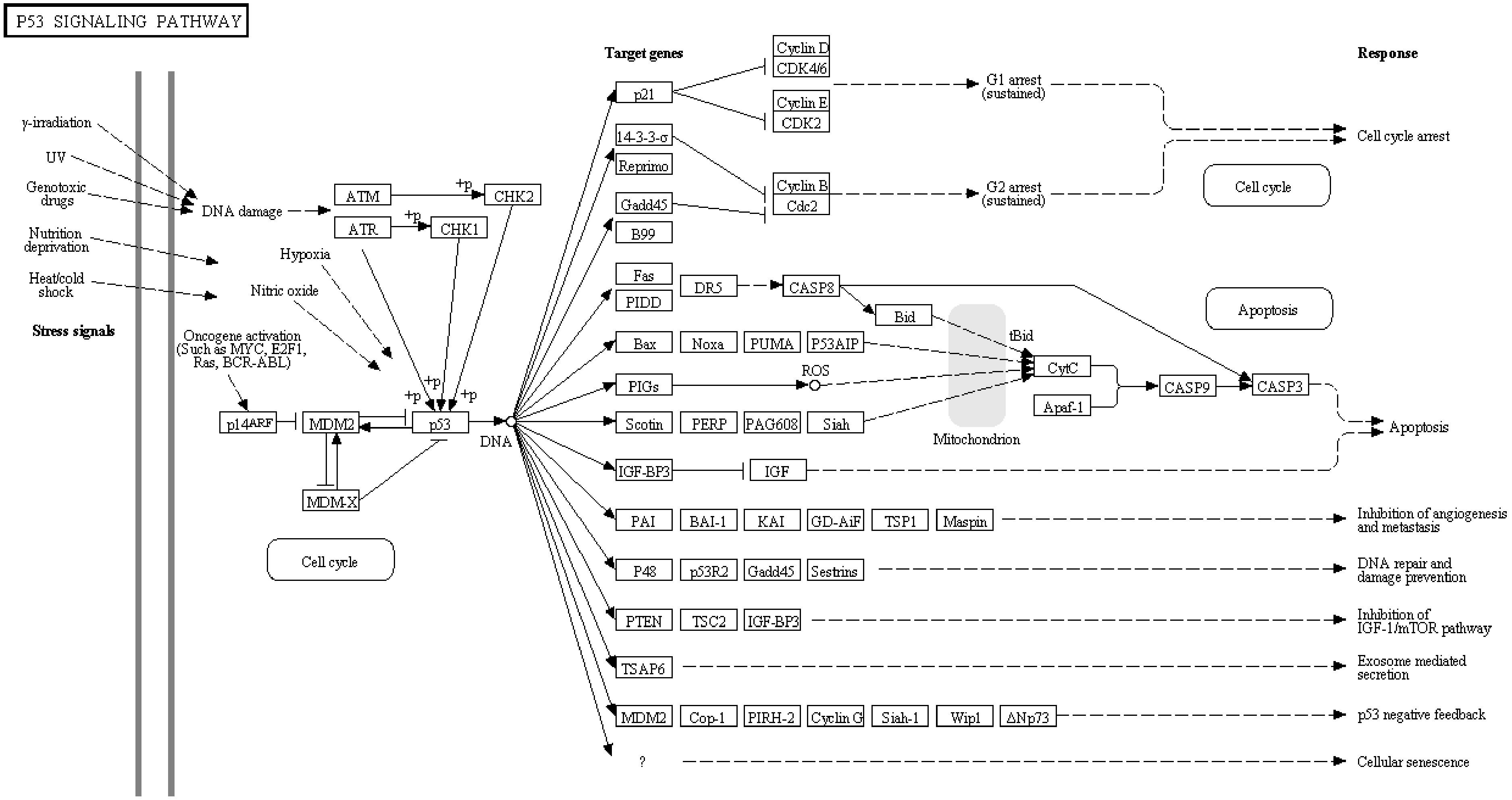

Figure S6. *Cont.*

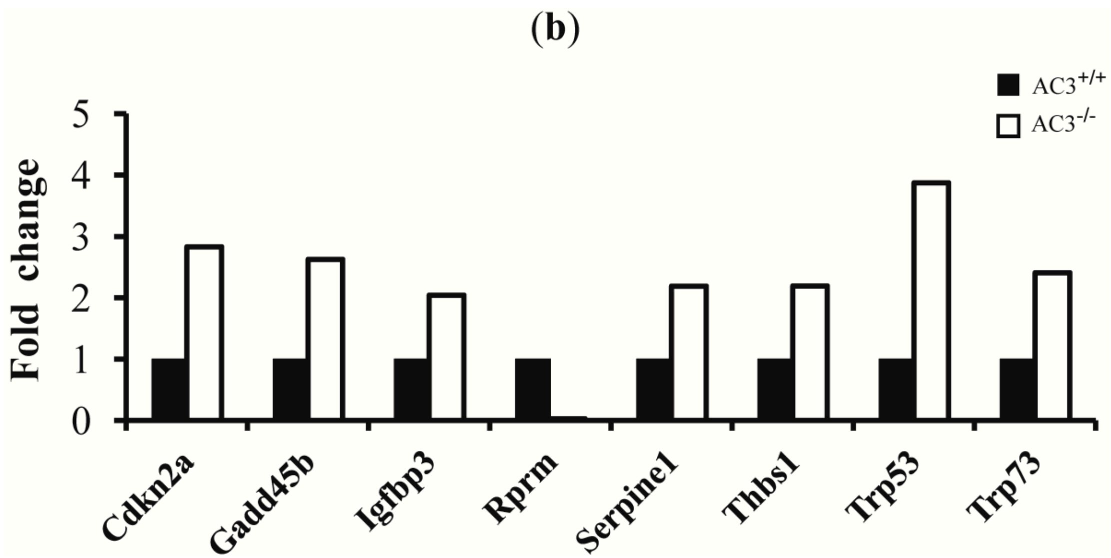

Figure S6. Expression levels of the genes in the P53 signal transduction pathway were altered after *AC3* deletion. (**a**) Influence of the differentially expressed genes on P53 signaling pathway and related signaling pathways; (**b**) The changes of the expression levels of the differentially expressed genes in P53 signaling pathway from the microarray results.
